# An Attempt to Classify Elementary Reactions on the Basis of TS Motifs

**DOI:** 10.1002/minf.202400040

**Published:** 2025-02-18

**Authors:** Kenji Hori, Yujiro Matsuo, Toru Yamaguchi, Kimito Funatsu

**Affiliations:** ^1^ TS Technology, Co. Ltd. CFM(R&D) Center Tokiwadai, Ube, Yamaguchi 755-8611 Japan; ^2^ Yamaguchi University Graduate School of Sciences and Technology for Innovation Ube 755-8611 Japan; ^3^ National Institute of Advanced Industrial Science and Technology Catalytic Chemistry Research Center Tsukuba 305-8560 Japan; ^4^ Nara Institute of Science and Technology Data Science Center Ikoma, Nara 630-0192 Japan

**Keywords:** kohonen map, QMRDB, self organization, transition state geometry, TS motif

## Abstract

Reactions commonly used in synthetic organic chemistry are named after their discoverers or developers. They are called the name reactions and generally consist of several elementary reactions. Quantum chemical calculations can optimize transition state (TS) structures of the elementary reactions. The geometrical feature of TS is called TS motif. We have constructed a database (QMRDB) with the TS motif information and have been continuing to accumulate them. In the present study, we extracted 102 elementary reactions from the QMRDB and attempted to classify them using the Kohonen self‐organization map. As the results, all the TS motifs were clustered. By firing a target compound on a Kohonen map generated, we expect to be able to easily find the TS motifs most similar to the target.

## Graphical abstract

The geometric characteristics of the TS structure are called TS motifs. In this study, we calculated 13 descriptors from the TS motifs and created Kohonen maps trained with these descriptors. As a result, we found that the obtained map could classify 102 elementary reactions.

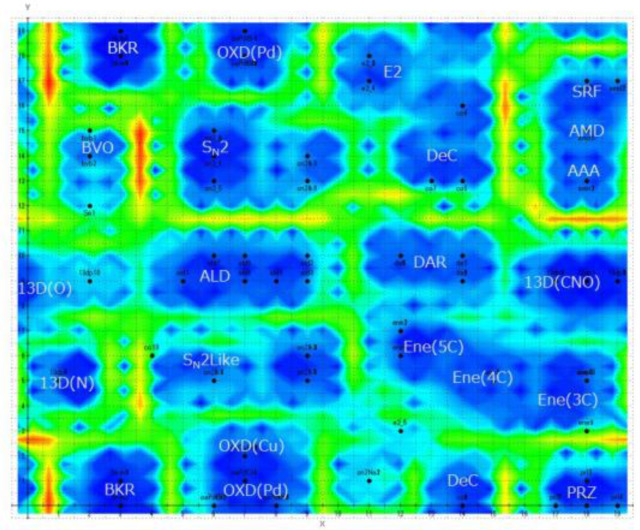

## 1 Introduction

Name reactions are often used when synthesizing new organic compounds [1]. Their reaction mechanisms have been investigated using experimental techniques in detail, and not only the transition state structures but the structural changes that accompany the progress of the reactions have been determined. According to the reaction mechanisms of name reactions, organic chemists can synthesize their targets. This experimental knowledge was confirmed by quantum chemical calculations, i. e., searching transition state structures, followed by intrinsic reaction coordinates (IRC) [2] calculations. These results mean that we can classify the name reactions on the basis of transition state structures. However, to the best of our knowledge, this type of classification has not been carried out so far because there has been no database with transition state structures for the many and diverse name reactions. In the present study, we attempted to classify elementary reactions, considering that most name reactions are composed of several elementary reactions.

**Figure 1:**
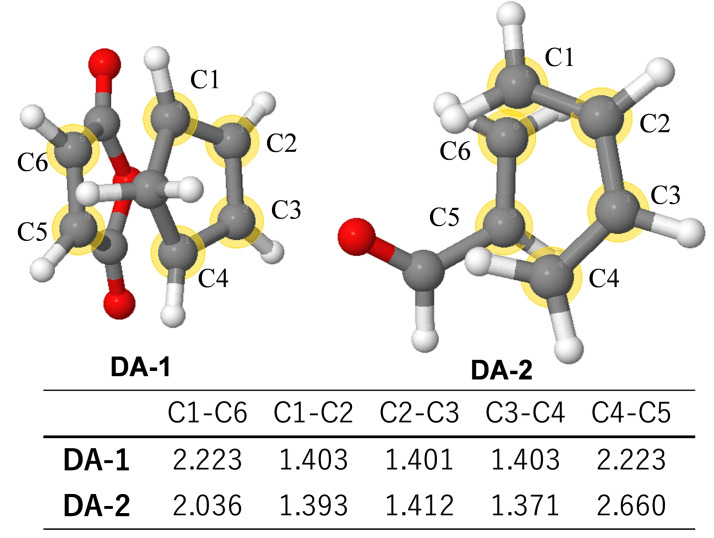
Similarity of TS motifs in Diels‐Alder reaction.

Our experience in investigating the mechanisms of many chemical reactions led to a conclusion that differences in substituents do not significantly change TS geometries for most elementary reactions [3]. This similarity is well appeared in the TS structures of the Diels‐Alder reaction shown in Figure 1.

**DA‐1** is the TS structure of cyclopentadiene and maleic anhydride and **DA‐2** that of butadiene and acrolein. While the two reactants of **DA‐1** have ring structures, **DA‐2** is constructed with a simple diene and an alkene with a formyl group. Although the nature of the substituents in the two TSs is different very much, both TSs have geometrical features that lead to the formation of six‐membered rings around C1‐C6. The differences in the C1‐C6 and C4‐C5 distances are relatively large (0.117 and 0.437 Å) since the number and the nature of the substituents are different. On the other hand, there are no large differences in the other distances (0.032 Å at most). We refer to the characteristic geometry of the reaction center as the TS motif, and the C1‐C6 atoms are called the TS motif atoms.

The similarity of TS motifs can be used not only to facilitate creating initial structures for TS calculations with different substituents, but also to reduce the calculation time required for TS optimization. This technique is called the TS motif method [4, 5]. The TS structure of a chemical reaction is clearly defined by Fukui′s IRC theory, and any structure that does not satisfy its conditions is not a TS structure. Therefore, TS structures optimized according to procedures such as the minimum energy path and the QST2 method available in the Gaussian09 program are the same as those optimized by the TS motif method within the limits of computational error, as long as the same level of theory is used.

In many cases, the TS motif method does not yield the most stable conformation in the TS structures. Therefore, we usually perform conformation analysis with the fixed TS motif moiety using the Conflex program [6], followed by performing TS optimizations at the B3LYP/6‐31G(d) level theory for at least the 10 stable conformations. The most stable one in the optimized structures is registered in TSDB [7, 8].

In addition, the results of previous TS conformation analyses revealed that the parameters of the TS motif (interatomic distances, bond angles, dihedral angles) do not change significantly even if the conformations of the substituents are different. In this study, we are using only the information from the TS motif moiety, and therefore, no significant changes may occur in the Kohonen map even if the TS motifs of the most stable TS conformation are not adopted.

In order to utilize this property for synthesis route developments of chemicals 9, we have been constructing a database (QMRDB) 10. The database stores a large number of calculated TS motifs for reactions which are frequently used in organic syntheses. For this reason, the QMRDB collects information on reactions commonly used in organic synthesis, and there is a relatively large amount of TS information for the 14 reactions used in this study. Furthermore, we randomly extracted several TS motifs from these elementary reactions, and the total number of data was 102. We created Kohonen maps that were trained using descriptors calculated from these TS motifs, and used it to classify reactions.

The QMRDB data contains TS information mainly at the B3LYP/6‐31G(d) or B3LYP/LANL2DZ level of theory from the Gaussian09 calculations 13. The database includes many data related to reactions of large molecules with more than 50 atoms. Therefore, the QMRDB data require a good balance between computation time and data accuracy. The theoretical level adopted for the database construction meets this requirement. The stored structure can also be used as initial structures for higher level TS calculations.

As the QMRDB has collected information on transition state coordinates, TS motifs and so on for more than 3,000 elementary reactions, we are now ready to try classifying reactions based on transition state structures. In the present study, we attempted to see if the features of TS motifs can be used to classify elementary reactions by using 13 descriptors of TS motifs as will be discussed later. For this purpose, we took advantage of the features of the Kohonen network: (1) it can maintain data adjacencies, and (2) it is easy to visualize clusters using interval distances indicated by U‐MATRIX.

## 2 TS Motifs and Their Nature

### 2.1 Descriptors for Self‐Organization

Many papers have been published on the analysis of reaction mechanisms using quantum chemical calculations. They discuss bond breaking and formation as the reaction proceeds, in relation to the TS structure. They also point out the importance of combination of transition metals and ligands on why, for example, catalytic reactions proceed. We have also published several papers discussing such issues and named the combination of geometric and qualitative features in TS structures as TS motifs. Therefore, when attempting to classify reactions using the results of theoretical reaction, the selection of TS motif features as descriptors is natural and a correct choice for classifying elementary reactions. The following descriptors of the TS motifs were extracted for making Kohonen self‐organization maps.

**Figure 2:**
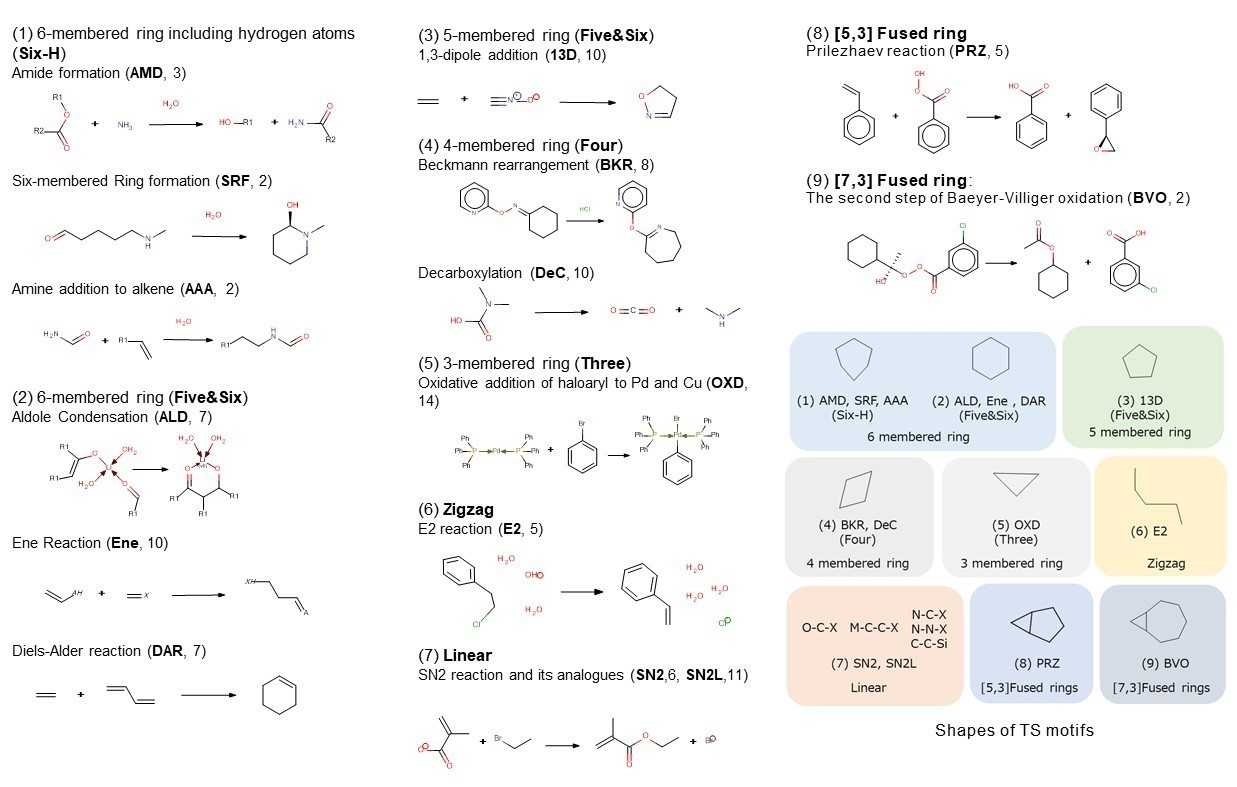
Elementary reactions extracted for the analysis and their types of TS motifs.

- Distance between a pair of atoms leading to form a new bond (**BF1**–**BF3**) and those leading to break a bond (**BB1**–**BB3**).
- Number of atoms consisting of the TS motif.
- Numbers of carbon, nitrogen, oxygen, hydrogen, halogen and typical/transition metal atoms in the TS motif.

i) is considered to be the descriptors of the geometrical or **quantitative** features of the TS motifs. Six distances were adopted as the descriptors to analyze reactions involving up to three **BB**s (**BB1**–**BB3**) and **BF**s (**BF1**–**BF3**), as shown in Table 1. For example, **DAR** in Figure 2 accompanies the formation of two bonds, but no bonds to be broken so that for **BF1** and **BF2**, we set their distance values, and for the other descriptors (**BF3** and **BB1‐BB3**), we set their values to 0.0. Since **SN2** involves one **BB** and one **BF**, the distance values were set to **BB1** and **BF1**, and 0.0 for the others (**BB2**, **BB3**, **BF2**, and **BF3**). The distances were ranked according to (a) the sum of the atomic numbers of the two atoms, and then, (b) the distance between the two atoms if the sum is the same. For **SRF** and **AAA**, one double bond becomes a single bond before and after reactions has completed. This change in the bonds is also included in BB. Appendix 1 shows a figure which shows **BF**s and **BB**s involving in the two reactions together with **AMD**.

ii) and iii) are considered to be the descriptors of qualitative properties of the TS motifs. The former is one of the factors that determine TS motif shapes shown in the right bottom of Figure 2. Furthermore, numbers and types of atoms constituting the TS motif is closely related to both the shape of the TS motifs and the difficulty in reactions to proceed. For example, atoms such as C, H, N, O, and halogens are involved in many organic reactions. In catalytic reactions, typical/transition metal atoms play important role in reactions to proceed. Therefore, the atom species and their numbers in the TS motifs are also employed as the descriptors.

In total, we employed 13 descriptors and did not consider any others. Therefore, we did not consider a large number of descriptors, nor did we select among them the descriptors that relate the shape of the TS motif to the TS motif descriptors combination (TMC).

As mentioned above, we extracted the 102 TS motifs for 14 types of the elementary reactions from the QMRDB. They include not only typical organic reactions, but reactions involving typical metals such as Li and Na as well as transition metals like Pd and Cu. Reactions with π‐bond breaking were not considered. Figure 2 displays the extracted elementary reactions for classifying the reactions using only descriptors that characterize the TS motifs. 102 TS data are randomly extracted a few from each elementary reaction. The number of each extracted reaction was given in parentheses along with the abbreviation. The abbreviations are used to indicate the position of each elementary reaction on the Kohonen map. All the descriptors for all the reactions are given in Appendix 2. All reaction equations for the self‐organization were summarized in Appendix 3.

### 2.2 Features of Reactions

The TS motifs for elementary reactions extracted from the QMRDB are classified into the following nine types as shown in the lower right of Figure 2. These features can be expressed as follows.

#### 2.2.1 Six‐H

Consider the reaction **SRF** in which a C‐N bond forms and the carbonyl group of the reactant is subsequently changed to a hydroxyl group of the product.

**Figure 3:**
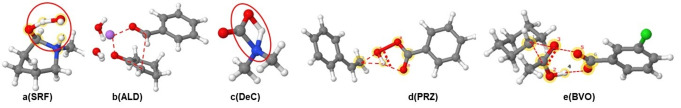
TS structures with characteristic TS motifs.

As shown in the red circle of **a(SRF)** in Figure 3, the amine hydrogen is transferred to the oxygen of the water molecule instead of the carbonyl oxygen, and the hydrogen of the water molecule is transferred to the carbonyl oxygen, i. e., the proton relay. The other two reactions (**AMD**, **AAA**) involve similar water‐mediated proton transfers. In protic solvents, the solvent itself or the water molecule in solvent mediates the proton transfer. Our experiences indicate that this involvement has the effect of reducing the activation free energy (ΔG^ǂ^) by ~10 kcal/mol in comparison with that of the direct one 14.

#### 2.2.2 Five&Six

Diels‐Alder reaction 15–17 produces a six membered ring due to two C‐C bond formation after the reaction is completed. The TS structure has a geometry that leads to form the ring as shown in Figure 1. A similar change is seen in **Ene**. 1,3 dipole addition (**13D**) 18 makes two C‐X (X‐O or N) bonds leading to the formation of a five‐membered ring. Although the TS of **b(ALD)** takes a six‐membered ring, it disappears in the product.

#### 2.2.3 Four

Beckmann rearrangement reaction (**BKR**) is considered to proceed via a nitronium ion 19. In the example shown here, this ion was optimized as a TS structure with a four‐membered ring geometry rather than the nitronium ion intermediate.

When the hydrogen of the carboxyl group is transferred to the amine nitrogen during decarboxylation, the reaction proceeds via the TS with a four‐membered ring, **c(DeC)**. The ΔG^ǂ^ value was calculated to be as low as 19.2 kcal/mol at the B3LYP/6‐31G(d) level of theory although the TS takes a four membered ring geometry.

#### 2.2.4 Three

The oxidative addition (**OXD**) is a reaction in which the carbon‐halogen bond of aryl halide is cleaved and the resulting two fragments coordinate to a transition metal such as Pd or Cu 20,21. The TS has a triangular geometry consisting of metal, carbon and halogen atoms.

#### 2.2.5 Zigzag

The E2 reaction (**E2**) often proceeds in competition with the S_N_2 reaction. The TS structure has a zigzag shape because the hydrogen that the nucleophile withdraws is bonded to the carbon adjacent to the carbon to which the leaving group is bonded.

#### 2.2.6 Linear

It is well known that the S_N_2 reaction (**SN2**) takes a linear TS geometry which leaving and nucleophilic groups are arranged at either side of the central carbon atom 22. Although the structures are similar, the reactions of **SN2L** are different in the types of atoms involved.

#### 2.2.7 [5,3] and [3, 9] Fused Rings

TSs of Prilezhaev reaction **d(PRZ)** 23 and Baeyer‐Villiger oxidation **e(BVO)** take fused ring geometries. The latter reaction consists of two elementary reactions. We extracted the second step TS, in which the ester is formed from an intermediate with the tetrahedral geometry.

The reactions in Figure 2 are characterized using TMC of qualitative ones. They are the number of atoms comprising a TS motif (ATOM#) and the number of bonds that are cleaved (BB#) or formed (BF#) before and after the reactions complete. Hereafter, the property will be denoted as TMC (Atom#, BF#, BB#) summarized in Table 1. This combination is the same for the reactions in **Six‐H**, but not for the other reactions. As will be discussed later, TMC is closely related to the shapes of the TS motifs.

**Table 1:** The TS motif descriptors combination (TMC).

Abbr.	Atom#	BF#	BB#
AMD	6	3	3
SRF	6	3	3
AAA	6	3	3
ALD	6	1	0
DAR	6	2	0
Ene	6	2	1
13D	5	2	0
BKR	5	2	2
DeC	4	1	2
OXD	3	2	1
SN2	3	1	1
SN2L	3	1	1
E2	3	1	2
PRZ	5	3	2
BVO	7	1	3

### 2.3 Program for Clustering the TS Motifs

**Figure 4:**
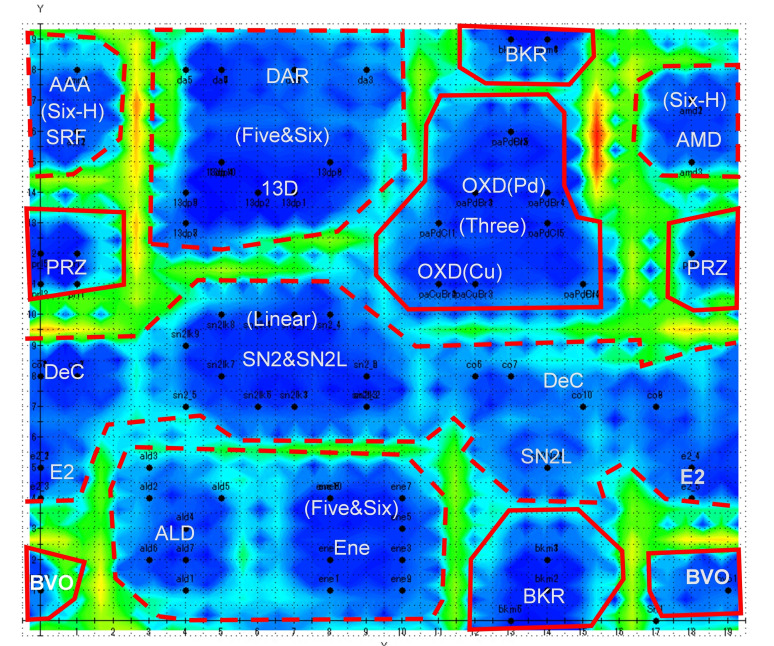
Kohonen Map based on quantitative descriptors.

**Figure 5:**
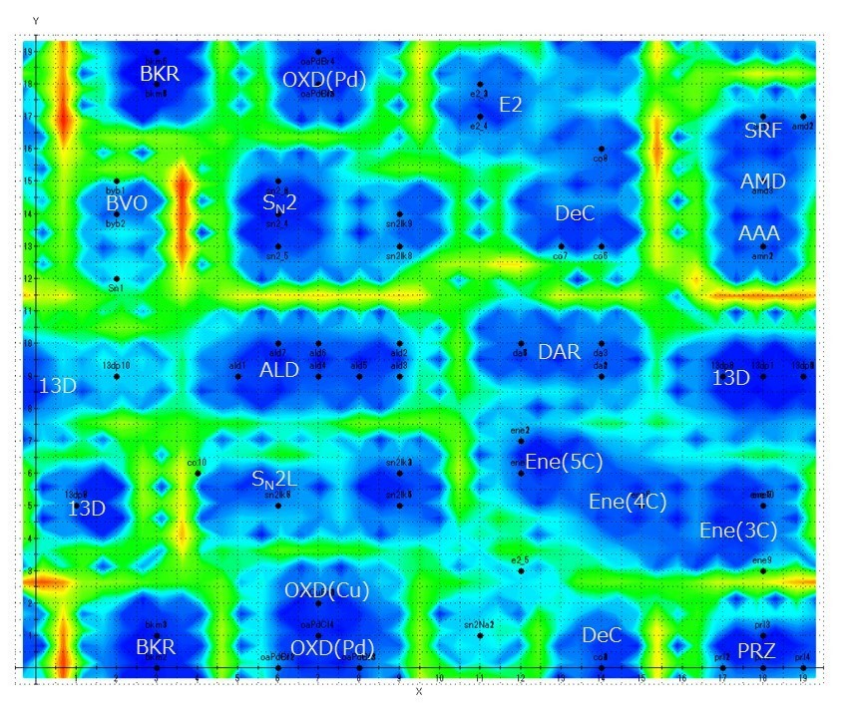
Kohonen Map of the TS Motifs based on the 13 descriptors.

The Chemish 5.09 program from Cheminfonavi was used to create the Kohonen maps 24. The conditions for making the self‐organizing map are: map size 20×20, number of learning sessions 50, learning range 5, and learning rate 0.03. Triangles were used as the nearest neighbouring function. Auto‐scaling and data shuffling were performed to classify the TS motifs. Furthermore, the distances between neurons (weight vectors) on the Kohonen map, which is an expanded torus map, was displayed as contour lines using the U‐Matrix method 25. Figures 4 and 5 show generated Kohonen maps showing the firing positions of 102 elementary reactions. On the map, the abbreviations such as **AMD**, **DAR** correspond to those for elementary reactions in Figure 2. Blue regions of the figures indicate self‐organized clusters. On the other hand, the red to yellow regions indicate the boundaries among clusters. The higher the boundary wall (i. e., the greater the distance between clusters), the darker the red.

## 3 Kohonen Map of Classifying TS Motifs

### 3.1 Classification Due to the Geometrical Descriptors

Figure 4 shows the Kohonen map only on the basis of the quantitative descriptors of the TS motifs. The reactions in the map are divided into three groups. The first consists of four independent reactions (**BKR**, **OXD**, **PRZ** and **BVO**) within solid lines. The second two groups include **Six‐H** and **Five&Six** surrounded by dashed lines. The third is that not explicitly separated. The three groups have the following characteristics.

#### 3.1.1 The Independent Reactions

##### 3.1.1.1 BKR

The reaction characterized by a four‐membered ring geometry in the TS and TMC (5,2,2), is separated from others. The reactions fires in the upper and lower right.

##### 3.1.1.2 OXD

Oxidative addition of transition metal complexes are characterized by TMC(3,2,1) and are located in the upper right as an independent group. The triangular structure and the involvement of transition metal atoms (Pd and Cu) lead **OXD** to the independent group distinct from organic reactions.

##### 3.1.1.3 PRZ and BVO

The TS structures of **d(PRZ)** and **e(BVO)** are characterized as [3, 6] and [3, 9] fused ring structures and have TMC (5,3,2) and TMC (7,1,3), respectively.

#### 3.1.2 The Group Reactions

##### 3.1.2.1 AAA, SRF and AMD

They are grouped as **Six‐H** with the same TMC (6,3,3) at the upper left and the upper right. The reactions accompany the proton relay of the two hydrogens as discussed above. As the red/yellow wall separates the region from the others, the cluster locates far in the Euclidean distance from the others. In other words, the mechanism of the reaction is very different from those of the others. Although the three reactions are classified in the same group, they fire in different positions on the map.

##### 3.1.2.2 DAR, 13D and Ene

Those with TMCs of (6,2,0), (5,2,0), and (6,2,1) are clustered in the upper left and the lower left as **Five&Six**. These reactions are classified as the pericycle reactions in organic chemistry. Although their TMCs are different, these reactions can be grouped together as the reactions via cyclic transition states.

##### 3.1.2.3 ALD

Although Aldole condensation of TMC (6,1,0) is in the same group, the TS structure **b(ALD)** is different from those of the other three reactions very much.

#### 3.1.3 The Unclassified Reactions

##### 3.1.3.1 SN2, SN2L, E2 and DeC

These reactions are unclassified. TMCs of the first two, **E2** and **DeC**, are represented as (3,1,1), (3,1,2), and (4,1,2), respectively. They locate in a band across the center of the figure and fire at different positions.

It is possible to roughly classify the 14 types of the elementary reactions using only the quantitative descriptors. Next, we attempted to generate the other type of Kohonen map by using quantitative as well as qualitative descriptors.

### 3.2 Self‐Organization with Additional Descriptors of the Qualitative Characters

The quantitative descriptors were not good enough to divide all the TS motifs to individual clusters. Therefore, we tried to make another Kohonen map considering the additional descriptors ii) and iii) of the qualitative properties. As shown in Figure 5, all the TS motifs could be identified.

#### 3.2.1 DAR, 13D and Ene

These reactions fire at the area in the right center although there are low walls among them, i. e., the Euclidean distances among them are small. We extracted **Ene** with three types of TS motifs such as **Ene(5c)**, **Ene(4C)**, and **Ene(3C)**, which differ in the atomic composition of the substrate as shown below. 
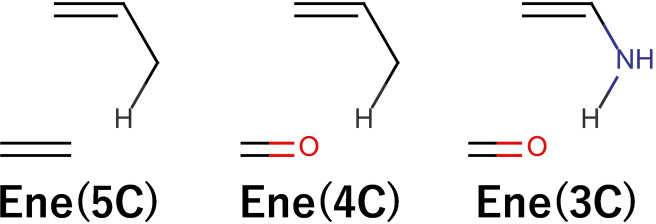


Although **Ene(5C)**, **Ene(4C)**, and **Ene(3C)** are Ene reactions, they fire at different locations on the map. The carbon atom receives a hydrogen atom in **Ene(5C)** and the oxygen atom in **Ene(4C)**. While the carbon atom donates a hydrogen atom in **Ene(4C)**, that in **Ene(3C)** is the nitrogen atom. The combination of the different descriptors was also important to distinguish among the three reactions.

They fire at different positions within the same cluster. In the case of these reactions, the TSs of the latter two reactions were optimized with reference to the TS of **Ene(5c)**. This result indicates that TS motifs clustered in the same region have geometrical similarities even if they have different reaction names and constituent atoms. Therefore, TS motifs used in digital screening do not necessarily have to have the same reaction name.

#### 3.2.2 E2 and DeC

The two reactions are clustered in the upper right center and the right bottom in Figure 5. They were regarded as the similar reactions. This result comes from the mechanism whereby a single molecule splits into two or three pieces. However, they were considered to be different type reactions since they fire and make clusters at different areas with low walls.

#### 3.2.3 ALD

The reactions with the TS geometry of **b(ALD)** form an independent group in the central region. This is very different from that seen in Figure 4. The TS has the characteristic TMC(6,1,0) and a Li ion. In the TS motif of **ALD**, a metal ion is considered to promote the progress of the reaction by coordinating the substrates, collecting them to the reaction center and activating them. Although TS motifs of **DAR**, **13D** and **Ene** have the six‐membered ring geometry, they do not have such specific metal ions to promote the reaction. This difference in the nature of the TS motifs is responsible for clustering the reactions with cyclic TS structures at different locations on the Kohonen map.

#### 3.2.4 SN2 and SN2L

The clusters of the two reactions are located in completely different regions near the center of the figure across **ALD**, despite they have similar TSs in geometry. The addition of the descripters such as the number and type of atoms comprising the TS motif makes a significant difference in the classification of the two types of the TS motifs.

## 4 Conclusions

We have accumulated a large number of TS motifs of elementary reactions that constitute name reactions. In this study, we extracted the 102 TS structures with the 14 different types of the elementary reactions and attempted to classify them using 13 descriptors with two different types of the properties.

102 reactions could only be classified by combining the distances of **BB**, **BF** (quantitative features) with the number and type of atoms comprising the TS motif (qualitative features). For example, **AMD**, **SRF**, and **AAA** with the same TMC were classified as almost the same category of reactions, but they fired at different positions on the map, i. e., they were distinguished each other. In other words, the mapping took into account qualitative features such as TMC as well as quantitative features such as **BF** and **BB**, resulting in a reaction classification. This result indicates that TMC only represents the similarity of the shape of TS motifs. The three reactions cannot be classified in detail without the combination of the different types of the descriptors.

It is interesting to ascertain whether or not a similar classification is possible for all reactions stored in the QMRDB. If the 13 descriptors are insufficient, we have to add other descriptors. For example, although not included in this study, P, S, and Si atoms are often included in TS motifs of organic reactions. In such reactions, their qualitative descriptors should be incorporated.

The present study revealed that it is possible to classify elementary reactions using the Kohonen maps trained with calculated descriptors from TS motifs. Reactions with different substituents, which belong to the same elementary reaction, fire in different positions on the map. We are currently investigating other possibilities for Kohonen maps trained with additional descriptors, i. e., the estimation of activation free energies without doing TS calculations for target reactions.

## Conflict of Interests

The authors declare no conflicts of interest.

5

## Data Availability

Research data are not shared.
